# Prevalence of dyslipidemia and its association with insomnia in a community based population in China

**DOI:** 10.1186/1471-2458-14-1050

**Published:** 2014-10-08

**Authors:** Yiqiang Zhan, Fen Zhang, Leihong Lu, Jinsong Wang, Yihong Sun, Rongjing Ding, Dayi Hu, Jinming Yu

**Affiliations:** Key Laboratory of Public Health Safety, Ministry of Education, School of Public Health, Fudan University, Shanghai, P. R. China; Institute of Clinical Epidemiology, School of Public Health, Fudan University, Shanghai, P. R. China; Department of Chronic Disease Prevention, Minhang Center for Disease Control and Prevention, Shanghai, P. R. China; Department of Dermatology, Linyi People’s Hospital, Linyi, P. R. China; Department of Preventive Medicine, School of Medicine, Yangzhou University, Yangzhou, P. R. China; Heart Center, Peking University People’s Hospital, Peking University, Beijing, P. R. China

**Keywords:** Insomnia, Dyslipidemia, Total cholesterol, Chinese population, Cross-sectional study

## Abstract

**Background:**

Studies of the prevalence of dyslipidemia and its association with insomnia are scarce in China. This study investigated the prevalence of dyslipidemia and its association with insomnia in a community based Chinese population.

**Methods:**

We conducted a cross-sectional survey in Beijing and recruited 10054 participants aged ≥18 years. The association between self-reported insomnia and dyslipidemia was determined by multiple logistic regression models. Age, gender, education, obesity, body mass index, physical activity, current smoking, current drinking, diabetes, and hypertension were adjusted as confounders. Odds ratios (ORs) with corresponding 95% confidence intervals (CIs) were reported as effect measurements.

**Results:**

The prevalence of dyslipidemia in those with no insomnia, occasional insomnia, and frequent insomnia were 53.3%, 54.3%, and 54.5% in men and 52.0%, 54.8%, and 61.2% in women. Compared with subjects with no insomnia, the multivariate adjusted ORs and 95% CIs for those with occasional insomnia and frequent insomnia were 1.07(0.86 ~ 1.34) and 1.19(0.89 ~ 1.60) for men, and 1.00(0.86 ~ 1.14) and 1.23(1.03 ~ 1.47) for women.

**Conclusions:**

These observations indicate that frequent insomnia was associated with a higher prevalence of dyslipidemia in women. This association was not significant in men.

## Background

Dyslipidemia is one of the most import and modifiable risk factors for atherosclerosis [1], stroke [2], and cardiovascular diseases [3]. Previous studies reported that the prevalence of dyslipidemia in China was lower than that in Western countries in the year around 2000 [4–9]. Nevertheless, China has been developing so rapidly during the last decades. The fast economic growth and social change are accompanied by transformation of daily lifestyle of the ordinary people. The prevalence of dyslipidemia might also have been changed and different from those in the previous reports. Thus, an update of our knowledge regarding the prevalence of dyslipidemia is largely on the agenda.

Studies suggested that sleep deprivation might be associated with dyslipidemia [10–12]. But few of them examined the association between insomnia and dyslipidemia in a population based survey. Insomnia is a subjective feeling of having difficulty initiating, maintaining or restoring sleep, or having poor sleep quality [13]. It was reported to be associated with substantial impairments of an individual’s quality of life [14], depression [15], and accident occurrence [16]. Although recent studies have showed that insomnia was associated with higher risks of cardiovascular diseases [17, 18], more research is needed to clarify its relationship with dyslipidemia. The primary objective of the present study as a priori was to test whether insomnia was associated with higher prevalence of taking into account of several established risk factors of dyslipidemia based on a community-based survey in a Chinese population.

## Methods

### Study design

This survey was a cross-sectional study regarding chronic diseases and risk factors conducted in Beijing in 2007 [19]. Citizens or permanent residents ≥18 years old were enrolled by a multistage stratified random sampling design. According to local economic development level, two urban districts, one urban–rural mixed district, and one rural district were selected, and then 38 communities (17 urban communities and 21 rural communities) were randomly sampled. Before collecting the data, we informed local administrators of the aim and method of our survey. With their help, we could convey information of our study design via broadcasting and booklets. At the night before our survey, the participants were told not to drink or eat from 8 pm to 8 am the next day. Written informed consents were obtained from all study participants, and the ethical approval was obtained from the Ethic Committee of Beijing Municipal Science and Technology Commission.

### Sample

In total, we intended to recruit around 300 participants in each study community. Finally, 12041 participants were invited, and 10054 participants were recruited with a response rate of 83.5%. Of them, 3687(36.7%) were men and 6367(63.3%) were women. The age of men in our sample ranged from 20 to 96 years with a median age being 53 years. Likewise, the age of women in this sample ranged from 20 to 91 years with a median age being 53 years.

### Data collection

The health interview was performed by trained medical staff at community health centers using a well-established and validated questionnaire to collect demographic and behavioral characteristics of the study population. Demographic information included birthday, gender, and education. Behavioral information included current smoking status, current drinking status, and physical activities. Education level was categorized as elementary school or lower (<7 years), middle or high school (7 ~ 12 years), and college or higher (>12 years).

Physical examination included anthropometric measurements, blood pressure, medical history, and drug administration history. Height and weight were measured to the nearest 0.1 cm and 0.1 kg respectively with the subject standing barefoot in light clothes. Waist circumference was measured to the nearest 0.1 cm at the mid-point between the 12th rib and right anterior superior iliac spine. Body mass index (BMI) was calculated as weight (kg) to be divided by square of height (m). Blood pressure was measured using standard mercury sphygmomanometer on the right arm in sitting position after the participants rested for 5 minutes. Phase 1 and phase 5 Korotkoff sound was used as systolic blood pressure (SBP) and diastolic blood pressure (DBP) respectively. Blood pressure was measured twice with the average results for the present data analysis. Medical history and drug administration history were obtained from medical records and confirmed by community general practitioners. All the measurements were adopted by community licensed physicians.

Blood samples were collected from all the participants after an overnight fasting. All the biochemical assessments were conducted in the central laboratory of Peking University People’s Hospital. Concentrations of fasting glucose, total cholesterol (TC), high-density lipoprotein cholesterol (HDL-C), triglycerides (TG), and low-density lipoprotein cholesterol (LDL-C) were measured using an auto analyzer (Hitachi 717, Hitachi Instruments Inc., Tokyo, Japan). Diabetes mellitus was defined as fasting glucose ≥7.0 mmol/L or current medication for diabetes [20]. Hypertension was defined as SBP ≥140 mmHg, DBP ≥90 mmHg, or current medication for hypertension [21]. Obesity was defined as waist circumstances >90 cm in men and >85 cm in women.

### Determination of self-reported insomnia and dyslipidemia

Self-reported insomnia was collected via a question: “During the last month, have you had insomnia (e.g.: difficulty falling asleep or non-restorative sleep)?” with the following response options: no insomnia, occasional insomnia (1–2 times/week), and frequent insomnia (≥3 times/week). Abnormal TC was defined as TC > 5.18 mmol/L, abnormal TG was defined as TG > 1.70 mmol/L, abnormal LDL-C was defined as LDL-C > 3.37 mmol/L, and abnormal HDL-C was defined as HDL-C < 1.04 mmol/L. Dyslipidemia was defined as any of the followings being abnormal: TC, TG, LDL-C, or HDL-C according to Chinese Guidelines on Prevention and Treatment of Dyslipidemia in Adults [22].

### Statistical analysis

Continuous variables were presented as mean ± standard deviation (SD) and categorical variables were presented as frequencies and proportions. In the descriptive analysis, we present the basic characteristics of study subjects and the prevalence of dyslipidemia and its individual components by insomnia. Cochran-Armitage method [23], which was developed for linear trends test in proportions or frequencies, was used for trend test in the associations between insomnia status (no insomnia, occasional insomnia, and frequent insomnia) and lipid status. Then in the exploratory analysis, we examined the association between insomnia and dyslipidemia using multiple logistic regression models in both men and women altogether. We also examined the interaction terms and found that there was an interaction effect between gender and insomnia for dyslipidemia. Then we examined the association in men and women separately. Three models were used for the analysis. The first model only included insomnia followed by the second model adjusted for age (plus gender for both genders together) as confounders. The third model was further adjusted for BMI, education, current smoking, current drinking, physical activity, obesity, diabetes, and hypertension as confounders. Odds ratios (ORs) with 95% confidence intervals (CIs) were presented, and *P* < 0.05 was considered to be statistically significant. All of the statistical analyses were conducted using R 2.15 [24].

## Results

### Basic characteristics of study subjects

**Figure 1:**
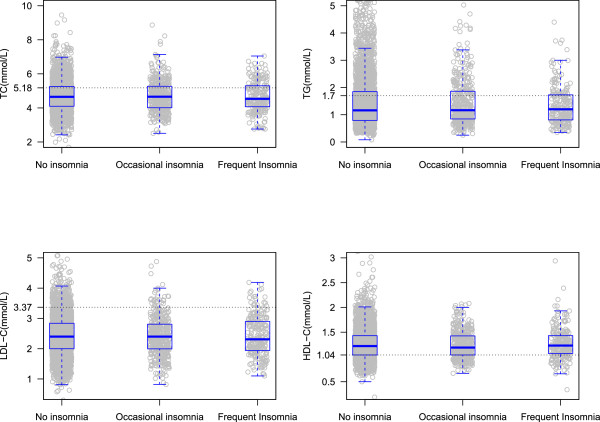
**Concentration of TC, TG, LDL-C, and HDL-C by insomnia in men.**

**Figure 2:**
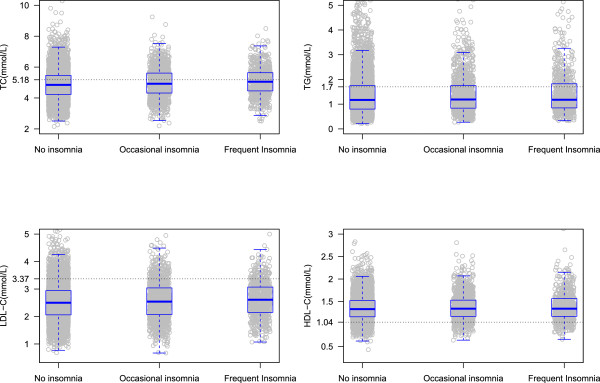
**Concentration of TC, TG, LDL-C, and HDL-C by insomnia in women.**

Table 1 demonstrates the basic characteristics of study participants by gender and insomnia status. In total, the numbers of men with no insomnia, occasional insomnia, and frequent insomnia were 3065(83.1%), 400(10.8%), and 222(6.1%), respectively, and those of women were 4567(71.7%), 1145(18.0%), and 655(10.3%). The average ages of the subjects with no insomnia, occasional insomnia, and frequent insomnia were 52.4 ± 13.6, 52.7 ± 12.9, and 56.4 ± 13.7 years for men, while those for women were 51.4 ± 13.2, 54.4 ± 12.4, and 56.5 ± 11.8 years, respectively. Of the 10054 participants, 5374(53.5%) were found with dyslipidemia. The concentrations of TC, TG, LDL-C, and HDL-C among different insomnia status were plotted in Figure 1 for men and Figure 2 for women.

**Table 1:** **Basic characteristics of the study subjects**

		Men			Women		
		No insomnia(n = 3065)	Occasional insomnia(n = 400)	Frequent insomnia(n = 222)	No insomnia(n = 4567)	Occasional insomnia(n = 1145)	Frequent insomnia(n = 655)
Age(years)		52.4 ± 13.6	52.7 ± 12.9	56.4 ± 13.7	51.4 ± 13.2	54.4 ± 12.4	56.5 ± 11.8
BMI(kg/m^2^)		25.1 ± 3.7	24.9 ± 3.7	24.5 ± 3.5	25.6 ± 4.1	25.4 ± 4.1	25.2 ± 3.9
TC(mmol/L)		4.71 ± 0.91	4.70 ± 0.92	4.73 ± 1.08	4.89 ± 0.98	4.98 ± 1.02	5.08 ± 1.00
TG(mmol/L)		1.64 ± 1.77	1.62 ± 1.65	1.47 ± 1.23	1.45 ± 1.24	1.48 ± 1.28	1.49 ± 1.11
LDL-C(mmol/L)		2.44 ± 0.67	2.43 ± 0.64	2.44 ± 0.70	2.54 ± 0.70	2.57 ± 0.71	2.62 ± 0.67
HDL-C(mmol/L)		1.27 ± 0.40	1.24 ± 0.28	1.26 ± 0.32	1.36 ± 0.29	1.37 ± 0.30	1.38 ± 0.32
Education, n(%)							
	0 ~ 6 years	663(21.6)	85(21.3)	62(27.9)	1398(30.6)	425(37.1)	291(44.4)
	7 ~ 12 years	2000(65.3)	256(64.0)	133(59.9)	2698(59.1)	625(54.6)	312(47.6)
	>12 years	402(13.1)	59(14.8)	27(12.2)	471(10.3)	95(8.3)	52(7.9)
Physical Activity, n(%)							
	No	1467(47.9)	206(51.5)	104(46.8)	2048(44.9)	495(43.3)	274(41.8)
	Yes	1598(52.1)	194(48.5)	118(53.2)	2516(55.1)	649(56.7)	381(58.2)
Current Smoking, n(%)							
	No	829(27.0)	109(27.3)	49(22.1)	4186(91.7)	1028(89.9)	565(86.3)
	Yes	2236(73.0)	291(72.7)	173(77.9)	381(8.3)	116(10.1)	90(13.7)
Current Drinking, n(%)							
	No	1504(49.1)	207(51.8)	113(50.9)	4351(95.3)	1088(95.0)	622(95.0)
	Yes	1561(50.9)	193(48.2)	109(49.1)	215(4.7)	57(5.0)	33(5.0)
Obesity, n(%)							
	No	1672(54.6)	221(55.3)	135(60.8)	2365(51.8)	585(51.1)	333(50.8)
	Yes	1393(45.4)	179(44.7)	87(39.2)	2202(48.2)	560(48.9)	322(49.2)
Work stress, n(%)							
	Low	391(12.8)	47(11.8)	31(14.0)	591(12.9)	119(10.4)	61(9.3)
	Intermediate	1695(55.3)	190(47.5)	84(37.8)	2547(55.8)	574(50.2)	291(44.6)
	High	978(31.9)	163(40.8)	107(48.2)	1428(31.3)	451(39.4)	301(46.1)
Diabetes, n(%)							
	No	2719(88.7)	351(87.7)	199(89.6)	4077(89.3)	1004(87.7)	567(86.6)
	Yes	346(11.3)	49(12.3)	23(10.4)	490(10.7)	141(12.3)	88(13.4)
Hypertension, n(%)							
	No	1824(59.5)	240(60.0)	130(58.6)	2957(64.7)	641(56.0)	326(49.8)
	Yes	1240(40.5)	160(40.0)	92(41.4)	1610(35.3)	504(44.0)	329(50.2)

### Prevalence of abnormal lipid profile

As shown in Table 2, the prevalence of dyslipidemia for those with no insomnia, occasional insomnia, and frequent insomnia were 53.3%, 54.3%, and 54.5% in men and 52.0%, 54.8%, and 61.2% in women. The Cochran-Armitage trend test for the prevalence of dyslipidemia across different insomnia status was *P* <0.0001 for all participants. These trend tests were not consistent in men (*P* = 0.6313) and women (*P* <0.0001).

**Table 2:** **Prevalence of dyslipidemia among subjects with different insomnia status [n(%)]**

		Abnormal TC	Abnormal TG	Abnormal LDL-C	Abnormal HDL-C	Dyslipidemia
All						
	No Insomnia	2457(32.2)	2100(27.5)	739(9.7)	1253(16.4)	4008(52.5)
	Occasional Insomnia	558(36.1)	431(27.9)	173(11.2)	214(13.9)	844(54.6)
	Frequent Insomnia	361(41.2)	238(27.1)	105(12.0)	128(14.6)	522(59.5)
P*		<0.0001	0.9525	0.0095	0.0203	<0.0001
Men						
	No Insomnia	845(27.6)	885(28.9)	242(7.9)	725(23.7)	1632(53.3)
	Occasional Insomnia	110(27.5)	125(31.3)	28(7.0)	98(24.5)	217(54.3)
	Frequent Insomnia	67(30.2)	57(25.7)	21(9.5)	52(23.4)	121(54.5)
P*		0.5001	0.7232	0.7058	0.9069	0.6313
Women						
	No Insomnia	1612(35.3)	1215(26.6)	497(10.9)	528(11.6)	2376(52.0)
	Occasional Insomnia	448(39.1)	306(26.7)	145(12.7)	116(10.1)	627(54.8)
	Frequent Insomnia	294(44.9)	181(27.6)	84(12.8)	76(11.6)	401(61.2)
P*		<0.0001	0.6173	0.0501	0.5673	<0.0001

*Cochran-Armitage trend test.

### Association of insomnia and abnormal lipid profile

Table 3 presents the association of serum lipid markers (TC, TG, LDL-C, and HDL-C) and insomnia. All of these models included age, BMI, education, physical activity, current smoking, current drinking, obesity, diabetes, and hypertension plus gender when men and women were analyzed together. ORs (95% CI) of TC for those with occasional insomnia, frequent insomnia were 1.00(0.79 ~ 1.27) and 1.15(0.85 ~ 1.57) compared with those with no insomnia in men. As for women, the ORs (95% CI) of TC were 1.04(0.91 ~ 1.20) and 1.24(1.05 ~ 1.49) for the participants with occasional insomnia and frequent insomnia. In terms of TG, LDL-C, and HDL-C, their associations with insomnia were not statistically significant. Additional analysis was made when treating serum lipids as continuous variables corresponding to Figures 1 and 2 by gender. We only observed significant results for TC in women (*P* <0.001).

**Table 3:** **Odds ratios and 95**% **confidence intervals between insomnia and TC, TG, LDL-C, and HDL-C**

		TC	TG	LDL-C	HDL-C
All					
	No Insomnia	1	1	1	1
	Occasional Insomnia	1.06(0.94 ~ 1.20)	1.03(0.91 ~ 1.18)	1.05(0.88 ~ 1.26)	0.96(0.81 ~ 1.13)
	Frequent Insomnia	1.25(1.07 ~ 1.45)	1.01(0.85 ~ 1.20)	1.07(0.86 ~ 1.34)	1.07(0.87 ~ 1.31)
Men					
	No Insomnia	1	1	1	1
	Occasional Insomnia	1.01(0.79 ~ 1.28)	1.20(0.94 ~ 1.54)	0.87(0.58 ~ 1.31)	1.07(0.83 ~ 1.39)
	Frequent Insomnia	1.17(0.86 ~ 1.58)	1.08(0.77 ~ 1.52)	1.19(0.74 ~ 1.92)	1.13(0.80 ~ 1.59)
Women					
	No Insomnia	1	1	1	1
	Occasional Insomnia	1.05(0.91 ~ 1.21)	0.92(0.79 ~ 1.08)	1.08(0.88 ~ 1.32)	0.86(0.69 ~ 1.07)
	Frequent Insomnia	1.25(1.05 ~ 1.50)	0.93(0.76 ~ 1.13)	1.03(0.80 ~ 1.33)	1.00(0.77 ~ 1.29)

TC, total cholesterol; TG, triglyceride; LDL-C, low density lipoprotein cholesterol; HDL-C, high density lipoprotein cholesterol.

Models for all: insomnia, age, education, obesity, BMI, physical activity, current smoking, current drinking, work stress, hypertension, and diabetes (plus gender when men and women analyzed together).

### Association of insomnia and dyslipidemia

The association of insomnia and dyslipidemia were described in Table 4. Model 1 included only insomnia, model 2 was adjusted for age (plus gender when men and women were analyzed together), and model 3 was further adjusted for several other confounders (BMI, education, physical activity, current smoking, current drinking, obesity, diabetes, and hypertension). Compared with those without insomnia, the corresponding ORs and 95% CI for subjects with occasional insomnia and frequent insomnia were 1.07(0.86, 1.34) and 1.19(0.89, 1.60) for men and 1.00(0.86, 1.14) and 1.23(1.03, 1.47) for women in the multivariable adjusted models. The ORs were not substantially different in the other models.

**Table 4:** **Odds ratios and 95**% **confidence intervals between insomnia and dyslipidemia**

		Model 1	Model 2	Model 3
All				
	No Insomnia	1	1	1
	Occasional Insomnia	1.08(0.98 ~ 1.21)	1.01(0.91 ~ 1.14)	1.06(0.94 ~ 1.19)
	Frequent Insomnia	1.33(1.15 ~ 1.53)	1.17(1.01 ~ 1.35)	1.28(1.10 ~ 1.49)
Men				
	No Insomnia	1	1	1
	Occasional Insomnia	1.04(0.84 ~ 1.28)	1.04(0.84 ~ 1.28)	1.08(0.87 ~ 1.36)
	Frequent Insomnia	1.05(0.80 ~ 1.38)	1.05(0.80 ~ 1.39)	1.21(0.90 ~ 1.63)
Women				
	No Insomnia	1	1	1
	Occasional Insomnia	1.12(0.98 ~ 1.27)	0.96(0.84 ~ 1.10)	1.00(0.87 ~ 1.15)
	Frequent Insomnia	1.46(1.23 ~ 1.72)	1.15(0.97 ~ 1.38)	1.25(1.04 ~ 1.50)

Model 1: insomnia.

Model 2: insomnia, age (plus gender when men and women analyzed together).

Model 3: insomnia, age, education, obesity, BMI, physical activity, current smoking, current drinking, work stress, hypertension, and diabetes (plus gender when men and women analyzed together).

## Discussion

Previous studies regarding sleep disorder or serum lipid were well documented in literature. Nevertheless, to our knowledge, few researches were conducted to explore insomnia and dyslipidemia simultaneously or examine the association between them, especially in the Chinese population [25]. In the present study, we investigated the association between self-reported insomnia and dyslipidemia and serum lipid markers in a community-based sample and found that frequent insomnia was related to higher prevalence of dyslipidemia and TC compared with those who reported no insomnia in women. This association was not statistical significant in men and independent of age, BMI, education, physical activity, current smoking, current drinking, obesity, diabetes, and hypertension.

Insomnia is usually accompanied with short sleep duration. Recent study suggested that short sleep duration was associated with higher risks of hypercholesterolemia in the adults of US [11] and Japan [12]. Likewise, reduced sleep duration was found to be related to dyslipidemia in other studies [26, 27]. However, some other studies reported different results which stated difficulty falling asleep was not associated with TG or HDL-C [28]. Different study design, source population, and socioeconomic status might explain the discrepancy. Previous studies also implied that women were more prone to suffer from insomnia than men [29, 30], and gender also played a key role in cardiovascular diseases and mortality [31]. Therefore, a gender inequality of the association between self-reported insomnia and dyslipidemia would be plausible. In our study, more women had insomnia than men (*P* < 0.0001). On the contrary, the prevalence of dyslipidemia in women was almost identical to that in men (*P* = 0.99). Thus, we hypothesized that the association between insomnia and dyslipidemia might be affected by gender. Our results did not contradict this hypothesis. The association of self-reported insomnia and dyslipidemia was significantly different between men and women. Insomnia might pose more risk to dyslipidemia in women. The reasons of the observed gender difference in the association between insomnia and dyslipidemia remains unclear, but recent research results were still able to provide suggestive clues. Firstly, it was reported that women were more prone to suffer psychiatric disorders and lacking coping ability [32, 33], both of which were related to sleep disorders. In addition to that, sex hormone was also found to be of paramount significance to exhibit influence on gender specific sleep habits [34]. Last but not least, lower socioeconomic status for women might partially explain the discrepancy [19]. In the present study, after adjusting for several well established risk factors for dyslipidemia, the association of insomnia and dyslipidemia was still significant in women. This indicated that other biological mechanisms might play a role in the gender differences. This research question should be addressed in future large longitudinal cohort studies.

In the present study, we also analyzed the association between insomnia and the individual components of serum lipid. We found that insomnia was associated with TC in women other than men, and we did not find a significant association between insomnia and TG, LDL-C, or HDL-C. This implied that the association between insomnia and dyslipidemia might be mainly due to the association of insomnia and TC. However, studies which directly explore the association of insomnia and serum lipid are scarce. Most of them focused on sleep duration and metabolic markers including serum lipid.

Several potential mechanisms might be proposed to explain the link of insomnia and dyslipidemia. Firstly, insomnia usually contributed to reduced sleep duration. People with reduced sleep duration tended to show a preference of high energy-density fatty food [35] and have a higher BMI via reducing leptin and elevating ghrelin [36, 37]. Higher BMI and intake of fat rich food were able to increase the risk of dyslipidemia [38]. In our study, the association of insomnia and dyslipidemia were still significant after adjusting BMI and obesity. This indicates that the observed association was independent of BMI or obesity. The link should be further explained by other possible mechanisms. Secondly, although the causes and physiology of insomnia have not been completely understood, it is generally considered as one kind of hyperarousal disorder related to increased activity of hypothalamic-pituitary-adrenal-axis [13]. During this process, cortisol [39] will be secreted excessively. Elevated cortisol is able to induce higher cholesterol [40]. Finally, Insomnia and dyslipidemia share some common risk factors, such as smoking [41, 42] and alcohol consumption [43]. Thus, the abnormalities of the neuroendocrine system and unhealthy lifestyle may present a biological plausibility between insomnia and abnormal lipid status. In addition to the aforementioned statements, depression or other psychiatric disorder might act as mediators in the association between insomnia and dyslipidemia. A meta-analysis using longitudinal epidemiological studies found that non-depressed individuals with insomnia had a twofold risk to develop depression compared with those without sleep difficulties [44]. Insomnia was also closely relatred to other psychiatric disorder [45]. Patients with major depressive or psychiatric disorders had a worse lipid profile in the Netherlands Study of Depression and Anxiety [46] as well as in a Chinese cohort [47]. In the present study, depression and psychiatric disorders were not measured, and further analysis regarding the mediating effects was unavailable. However, the mediation effects of depression and psychiatric disorders deserve detailed examination in future studies.

Our results have meaningful implications for both public health and clinical practice professionals. Since insomnia is a modifiable risk factor for chronic diseases including abnormal serum lipid profile, treating insomnia might have protective effects on lowering TC levels as well as deceasing dyslipidemia prevalence. However, more longitudinal studies and clinical trials are warranted to examine the causal relationship between insomnia and dyslipidemia or whether treating insomnia with medicine has beneficial effects on patients’ lipid levels.

This study has some limitations. The present data was collected using a cross-sectional design, we cannot make causal inferences regarding whether insomnia precedes or causes dyslipidemia. Furthermore, more women than men participated in the survey. Women paid much more attention to their health, which could result in an overestimation of the prevalence of insomnia in the source population. But this would have little effect on the reported association of insomnia and dyslipidemia after stratifying analysis by gender. Finally, insomnia was collected in a single question rather than multiple questions proposed by other studies [13], so we cannot examine the association between other insomnia symptoms and dyslipidemia in the present study.

## Conclusions

In conclusion, self-reported insomnia was associated with higher prevalence of dyslipidemia and TC in this large Chinese women sample. Future longitudinal studies should be conducted to further explore the causal relationship and to clarify the biological mechanisms between them.

## Abbreviations

TC: Total cholesterol
TG: Triglyceride
LDL-C: Low density lipoprotein cholesterol
HDL-C: High density lipoprotein cholesterol
BMI: Body mass index
SBP: Systolic blood pressure
DBP: Diastolic blood pressure
OR: Odds ratio
CI: Confidence interval.
